# Prevalence of human papillomavirus in archival samples obtained from patients with cervical pre-malignant and malignant lesions from Northeast Brazil

**DOI:** 10.1186/1756-0500-3-96

**Published:** 2010-04-08

**Authors:** José V Fernandes, Rosely V Meissner, Maria GF Carvalho, Thales AAM Fernandes, Paulo RM Azevedo, João S Sobrinho, José CM Prado, Luisa L Villa

**Affiliations:** 1 Department of Microbiology and Parasitology, Federal University of Rio Grande do Norte, Av. Sen. Salgado Filho, S/N, Lagoa Nova, CEP: 59072-970, Natal, RN, Brazil; 2 Department of Pathology, Federal University of Rio Grande do Norte, Av. Gal. Gustavo Cordeiro de Farias, S/N, Petrópolis, CEP 59010-180 Natal, RN, Brazil; 3 Department of Biomedical Sciences, University of Rio Grande do Norte State, Rua Miguel Antônio da Silva Neto, S/N, CEP: 59607-360, Mossoró-RN, Brazil; 4 Department of Statistics, Federal University of Rio Grande do Norte, Av. Sen. Salgado Filho, S/N, Lagoa Nova, CEP: 59072-970, Natal, RN, Brazil; 5 Ludwig Institute for Cancer Research, Hospital Alemão Oswaldo Cruz, Rua João Julião, 245, 1° Andar, Paraíso, CEP 01323-903, São Paulo-SP, Brazil

## Abstract

**Background:**

Human Papillomavirus (HPV) is considered as a necessary, but not sufficient, cause of cervical cancer. In this study, we aimed to assess the prevalence of HPV in a series of pre-malignant and malignant cervical lesion cases, to identify the virus genotypes, and to assess their distribution pattern according to lesion type, age range, and other considered variables. The samples were submitted to histopathological revision examination and analysed by polymerase chain reaction (PCR) for the presence of HPV DNA, followed by HPV typing by dot blot hybridisation.

**Findings:**

Of the analysed samples, 53.7% showed pre-malignant cervical lesions, and 46.3% presented with cervical cancer. Most cancer samples (84.1%) were classified as invasive carcinoma. The mean age of these cancer patients was 47.3 years. The overall HPV prevalence was 82.4% in patients with pre-malignant lesions and 92.0% in the cancer patients. HPV 16 was the most prevalent type, followed by HPV 18 and 58, including both single and double infections. Double infection was detected in 11.6% of the samples, and the most common combination was HPV 16+18.

**Conclusions:**

Cervical cancer appears to occur in women in a lower age range in the studied area, compared to the situation in other Brazilian regions. Furthermore, among the patients with CIN 3 and those with cancer, we observed a higher proportion of married women, women with more than one sexual partner, smokers, and individuals with less than an elementary education, relative to their counterparts.

**Findings:**

The overall HPV prevalence was 82.4% in patients with pre-malignant lesions and 92.0% in the cervical cancer patients from Northeast Brazil. HPV 16 was the most prevalent type, followed by HPV 18 and 58. The most common double infection was HPV 16+18. Cervical cancer appears to occur in women in a lower age range in the Northeast Brazil. Among the patients with CIN 3 and those with cancer, we observed a higher proportion of married women, women with more than one sexual partner, smokers, and individuals with less than an elementary education, relative to their counterparts.

## Background

Squamous cell cervical carcinoma is the third most common malignancy among women around the world, especially in underdeveloped countries, and it is the second cause of cancer death in women worldwide [1-3]. It is now widely accepted that human papillomavirus (HPV) infection of the cervical epithelium is a necessary, but not sufficient, cause of the development of cervical cancer, as well as a significant proportion of other anogenital and oral squamous cell carcinomas [4-6]. More than 120 different HPV types have been catalogued so far, and about 40 can infect the epithelium of the anogenital tract and other mucosal areas of the body. At least 15 of them, called oncogenic or high-risk HPV (HR-HPV), are strongly associated with progression to invasive cervical cancer [5,7,8].

In Brazil, the occurrence of cervical cancer continues to be a serious public health problem, showing remarkable differences in incidence among different regions of the country [9,10]. Archival formaldehyde-fixed, paraffin-embedded tissues are an important source for retrospective studies of HPV detection to contribute further to the implementation of public health measurements for the management and control of cervical cancer [11,12].

We have analysed a large series of cervical specimens of archival formaldehyde-fixed, paraffin-embedded tissues from women in a high-risk area for cervical cancer in northeastern Brazil. The samples were re-examined, and the lesions were histologically classified and analysed by polymerase chain reaction (PCR) for the presence of HPV, followed by HPV typing by dot blot hybridisation.

## Methods

A series of pre-malignant and malignant samples obtained from archival paraffin-embedded tissues were analysed. Initially, we selected specimens that had been collected between January 1996 and December 2000 from patients with a previous diagnosis of cervical lesions, treated at Luis Antonio Hospital, a cancer referral centre in the city of Natal, State of Rio Grande do Norte (RN), Brazil. Six serial 5-μm sections from each block were prepared: one was used for histopathological revision examination, while the remaining samples were processed for HPV DNA detection. After histopathological revision, we selected 215 samples diagnosed with severe cervical intraepithelial neoplasia (CIN 3) or cervical cancer, among which 9 cases were excluded due to the lack of representative neoplastic tissue in the block after histopathological evaluation, and 16 more were excluded due to the lack of amplifiable DNA, leaving a total of 190 samples for this study.

This study was approved by the Ethical Committee in Research at the Federal University of Rio Grande do Norte.

### DNA extraction

DNA was extracted as previously described by Banerjee et al. (1995) [13], with some minor modifications, including the substitution of Triton X-100 with Tween 20 in the digestion buffer.

### HPV DNA detection and typing

DNA samples were submitted to PCR analysis with the β-globin primers, PCO3/PC04 [14]. The resulting 110-bp amplicon served as an internal control to evaluate the integrity and sufficiency of the DNA extracted from cervical specimens. For HPV DNA detection, the generic primers, GP5+/GP6+, were used to amplify a fragment of about 140 bp [15]. PCR products were typed by dot-blot hybridisation, according to the method of Manos et al. (1989) [16].

### Statistical analyses

To verify the association between HPV prevalence and the considered variables, the χ^2 ^test and the proportion comparison test were performed using the Statistica 7.0 software. Values of p ≤ 0.05 were considered as statistically significant.

## Results

The presence of cervical lesions was confirmed in 206 of the 215 samples analysed by histopathological revision exam; these samples were then submitted to DNA extraction for molecular analysis. Among all of the samples, 190 were positive for the amplification of a human β-globin gene segment. Among them, 53.7% showed pre-malignant lesions classified as severe CIN 3; 38.9% presented with invasive squamous cell carcinoma (SCC); and 7.4% were classified as adenosquamous carcinoma (ADSQ).

Among the samples with only malignant lesions, most (84.1%) were histologically classified as SCC, whereas only 15.9% were categorised as ADSQ. We verified that the majority of the samples (60.0%) were obtained from non-Caucasian women (African-mixed-descendant) considered as non-white in this study, whereas 40.0% of the samples were from Caucasians considered as white. The age varied from 19 to 69 years, with a mean age of 44.3 ± 11.25 years for patients with CIN 3 and 47.3 ± 10.9 years for those with cervical cancer. Most of the CIN 3 samples (61.7%) were obtained from women between 19 and 47 years of age, while most of the cervical cancer samples (86.4%) were obtained from patients between 38 and 57 years of age (Table 1).

**Table 1:** Distribution of HPV prevalence according to the lesion types and considered variables.

	CIN 3 (n = 102)		Cervical cancer (n = 88)	
		
Variables	No. of Samples (%)	No. of samples with HPV (%)	No. of samples (%)	No. of samples with HPV (%)
**Age at study entry**				
≤ 37	14 (13.7)	11 (78.6)	14 (15.9)	13 (92.9)
38 - 47	49 (48.0)	41 (83.7)	40 (45.5)	37 (92.5)
48 - 57	28 (27.5)	23 (82.1)	22 (25.0)	20 (90.9)
≥ 57	11 (10.8)	9 (81.8)	12 (13.6)	11 (91.7)
**Ethnicity**				
White	45 (44.1)	37 (82.2)	31(35.2)	29 (93.5)
Non-white	57 (55.9)	47 (82.5)	57(64.8)	52 (91.2)
**Marital status**				
Single	30 (29.4)	25 (83.3)	29 (33.0)	27 (93.1)
Married	61 (59.8)	50 (82.0)	52 (59.0)	48 (92.3)
Other	11 (10.8)	9(81.8)	7 (8.0)	6 (85.7)
**Education**				
Less than elementary	56 (54.9)	46 (82.1)	49 (55.7)	45 (91.8)
Elementary	40 (39.2)	33 (82.5)	34 (38.6)	31 (91.2)
High school	6 (5.9)	5 (83.3)	5 (5.7)	5 (100.0)
**More than one partner**				
Yes	70 (68.6)	58 (82.9)	58 (65.9)	54 (93.1)
No	32 (31.4)	26 (81.2)	30 (34.1)	27 (90.0)
**Smoking**				
Yes	61 (59.8)	50 (82.0)	56 (63.6)	52 (92.9)
No	41 (40.2)	34 (82.9)	32 (36.4)	29 (90.6)
**HPV Positive**		**84 (82.4)**		**81 (92.0)**
HPV Negative		18 (17.6)		7 (8.0)
**Total**	**102 (100)**		**88 (100)**	

CIN - cervical intraepithelial neoplasia

In patients with CIN 3 or cancer, we observed a higher proportion of married women (p = 0.000, p = 0.001), women with more than one sexual partner in their lifetime (p = 0.000, p = 0.000), smokers (p = 0.010, p = 0.001), and individuals with less than an elementary education (p = 0.039, p = 0.023), relative to their counterparts. Moreover, we observed a higher incidence of cancer among the non-white women (p = 0.002) (Table 1).

Of the 190 samples positive for β-globin, 165 were also positive for the amplification of a target sequence of the HPV genome, revealing an overall HPV prevalence of 86.8% (Table 2).

**Table 2:** Distribution of HPV genotypes according to lesion types

	Lesion Types		
	
	CIN 3	SCC	ADSQ
	
HPV Genotype	No. (%)	No. (%)	No. (%)
16	53 (52.0)	45 (60.8)	2 (14.3)
58	5 (4.9)	7 (9.5)	
18	3 (2.9)	2 (4.1)	7 (50.0)
45	2 (2.0)	2 (2.7)	2 (14.3)
31	2 (2.0)	2 (2.7)	
33	2 (2.0)	2 (2.7)	
52	1 (1.0)	1 (1.3)	
59	2 (2.00	1 (1.3)	
16 + 18	5 (4.9)	1 (1.3)	2 (14.3)
31 + 33	3 (2.9)	2 (2.7)	
16 + 31	3 (2.9)	1 (1.3)	
16 + 52	1 (1.0)	1 (1.3)	
58 + 56	1 (1.0)	1 (1.3)	

**Positive**	**84 (82.4)**	**68 (91.9)**	**13 (92.9)**
Negative	18 (17.6)	6 (8.1)	1 (7.1)
Total	102 (100)	74 (100)	14 (100)

CIN - Cervical intraepithelial neoplasia, SCC - Squamous cell carcinoma, ADSQ - Adenosquamous carcinoma

Among the HPV-positive samples, 86.7% had a single infection, while 13.3% had a double infection. When each lesion type was considered separately, the observed HPV prevalence rate was 82.4% for CIN 3, 91.9% for SCC, and 92.9% for ADSQ. When both single and double infections were considered, HPV 16 was the most common HPV type, with a prevalence rate of 60.5% for overall patients, 61.8% for CIN 3, 64.9% for SCC, and 28.6% for ADSQ, followed by HPV 18 with a prevalence rate of 10.5% for overall patients, 7.8% for CIN 3, 4.1% for SCC, and 64.3% for ADSQ. HPV 58 followed with a prevalence rate of 7.4% for overall patients, 5.9% for CIN 3, 10.8% for SCC, and 0.0% for ADSQ. HPV double infection was detected in 11.6% of the samples, with a prevalence rate of 13.7% for CIN 3, 8.1% for SCC, and 14.3% for ADSQ. The most common combination of HPV double infections was HPV 16+18. When only the samples with cervical cancer, including single and double infections, were considered, the distribution of the most common HPV type in terms of prevalence rate, in decreasing order, was as follows: HPV 16, followed by HPV 18, 58, 31, 45, and 33 (Table 2).

We found statistically significant differences in the prevalence rates of HPV 16 and HPV 18 between the samples with CIN 3 and those with ADSQ (χ^2 ^= 24.76, p < 0.001). Similar differences were found between the samples with SCC and those with ADSQ (χ^2 ^= 27.29, p < 0.001) (Table 3).

**Table 3:** Distribution differences of the genotypes, HPV 16 and HPV 18 in CIN 3 and cervical cancer, including single and multiple infections.

				HPV 16	HPV 18	Χ^2 ^test	p
				
Diagnosis	N	Positive	Negative				
**CIN 3**	102	84	18	63	8	24.76	0.000*
**SCC**	74	68	6	48	3	27.29	0.000*
**ADSQ**	14	13	1	4	9	-	-

CIN - Cervical intraepithelial neoplasia, SCC - Squamous cell carcinoma, ADSQ - Adenosquamous carcinoma *Statistically Significant

## Discussion

The majority of the samples with cervical malignant lesions analysed in this study (84.1%) were histologically classified as SCC, while ADSQ was found in the remaining 15.9% of cases, a ratio similar to that described by the National Institute of Cancer, Brazil [10]. Considering that the specimens used in this study were fixed in non-buffered formalin, a 92.2% yield of amplifiable DNA can be considered as satisfactory.

The average age of the cervical cancer patients in this study was 47.3 years, which is lower than that of Brazilian women from other regions of the country, such as central (49 years) [9], northern (51 years) [17], and southeastern (52 years) Brazil [18]. This mean age of cervical cancer occurrence was higher than the reported average for six African countries (33.9 years) but was lower than that for three European countries (56.5 years) [1]. We observed that 61.4% of the patients with cervical cancer were up to 47 years of age and 15.9% were up to 37 years of age. This relatively high frequency of cervical cancer in young women could be due to the acquisition of high-risk HPV infection at a young age among the local female population. This hypothesis is in agreement with a previous study of the Natal-RN population conducted by Fernandes et al., (2008) [19] and a systematic review written by Smith et al. (2007) [20]. It is also likely that there are some high-risk HPV variants with an increased oncogenic potential circulating in northeast Brazil. Other factors that might also contribute to the low mean age of the cervical cancer patients found in this study are a low degree of education, a low socio-economic status, sexual behaviour, and failure of the screening programmes in preventing and controlling cervical cancer.

We observed a high incidence of cervical cancer in the non-white women. However, due to the high degree of interbreeding in the population of Rio Grande do Norte [21], the impact of ethnicity on the results of this study is difficult to assess.

We also found a high incidence of cervical cancer in married women, women with a low degree of education, women with more than one sexual partner in their lifetime, and smokers.

The overall HPV prevalence in cancer samples in this study was 92.0%, higher than that described for women from Goiânia, in the central region of Brazil (80.3%) [9]. Six types of HPV, all high-risk, were identified. HPV 16 was the most prevalent type in the samples with CIN 3 and SCC. HPV 18 was the most prevalent type in those with ADSQ. These results are consistent with those described in the literature [20,22,23].

Comparing the distribution of HPV types by prevalence order with that in other regions of Brazil, as described by Rabelo-Santos et al. (2003), the overall HPV 16 prevalence rate found in the current study (59.1%) was similar to that described for the central region (57.1%), almost equal to that described for the northeastern region (59.3%), but higher than that in the southern (52.2%), southeastern (53.8%), and northern regions (43.5%). The HPV 18 prevalence was higher than that reported for all other regions of the country, including the northeastern region. With regard to HPV 31, the prevalence rate observed in the present study was higher than that described for the central region, similar to that in the southern and southeastern regions, and lower than that described for the northeastern region. The HPV 33 prevalence rate found in this study was lower than that reported for the central, southern, and southeastern regions but similar to that for the northeastern region. However, these results of genotype distribution might also be influenced by the HPV genotyping techniques used in the different studies.

When only considering cancer cases and by comparing the prevalence rates of HPV types with a consistent worldwide study conducted by Bosch et al., 1995 [1], we found that the HPV 16 prevalence rate in the current study was similar to that found in Brazil (52.2%), higher than the related mean of six African countries (42.5%) and that of six South American countries (50.4%), and lower than the mean described for three European countries (65.1%). HPV 18 was second in prevalence, found in 13.6% of the cancer samples; its prevalence rate was higher than that of Brazil (8.7%), as well as that of South American (8.8%) and European (8.1%) countries, but it was lower than the prevalence rate of the African countries (17.7%). HPV 58 was detected in 9.1% of the samples, with a prevalence rate three times higher than the mean of the African countries (2.7%), four times higher than the mean of the Central and South American countries (2.2%), and seven times higher than the mean of the European countries (1.2%). A high HPV 58 prevalence was also described in patients with cervical pre-malignant lesions in the southern region of Brazil [24].

Geographical differences in HPV distribution of cervical cancer have recently been compiled [2,8] and serve as the basis for the implementation of cervical cancer control strategies around the world [25].

## Conclusions

Cervical cancer appears to occur in a lower age-range in women in this study, when compared with studies conducted in other Brazilian regions. Among the patients with CIN 3 and cancer, we observed a higher proportion of married women, women with more than one sexual partner, smokers, and individuals with less than an elementary education, relative to their counterparts.

## Competing interests

The authors declare that they have no competing interests.

## Authors' contributions

JVF conceived and coordinated the study, participated in its design, carried out the molecular analysis, and drafted the manuscript; RVM participated in the molecular analysis; MGFC carried out the histological analysis; TAAMF participated in the molecular analysis and helped to draft the manuscript; PRMA performed the statistical analysis; JSM and JCMP carried out the HPV typing; and LLV participated in the design and coordination of the study. All authors read and approved the final manuscript.
